# Elevated granulocytic myeloid-derived suppressor cells are closely related with elevation of Th17 cells in mice with experimental asthma

**DOI:** 10.7150/ijbs.43596

**Published:** 2020-05-16

**Authors:** Fei Xue, Mengzhu Yu, Li Li, Wenzhe Zhang, Yongbin Ma, Liyang Dong, Wenqi Shan, Yu Zheng, Ting Wang, Dingqi Feng, Jianping Lv, Xuefeng Wang

**Affiliations:** 1 Department of Central Laboratory, The Affiliated Hospital of Jiangsu University, Zhenjiang 212001, China; 2 Department of Pediatrics, The Affiliated Hospital of Jiangsu University, Zhenjiang 212001, China; 3 Department of Neurology Laboratory, Jintan Hospital, Jiangsu University, Jintan 213200, China; 4 Department of Nuclear Medicine and Institute of Oncology, The Affiliated Hospital of Jiangsu University, Zhenjiang 212001, China

**Keywords:** granulocytic myeloid-derived suppressor, Th17 cells, experimental asthma

## Abstract

Asthma is a complex and heterogeneous inflammatory response characterized by various immune cells, including myeloid-derived suppressor cells (MDSCs) and CD4^+^ T-cell subsets. However, few studies on MDSC subsets and the association between MDSCs and CD4^+^ T-cell subsets in asthma are reported. In the present study, we detected CD4^+^ T cells and MDSC subsets and evaluated the relationship of these cells in mice with ovalbumin-induced asthma. We found that asthmatic mice showed severe airway inflammatory response and inflammatory cell infiltration in the lungs and bronchoalveolar lavage fluid. We also noted increased numbers of Th2, Th17, and MDSCs; decreased proportion of Th1 and Treg cells in the splenocytes and lungs; and increased expression of pro-inflammatory cytokines in splenocytes and lungs. Granulocytic MDSCs (G-MDSCs) and Th17 cells were closely related. Gemcitabine treatment reduced the G-MDSC level and the iNOS expression, alleviated the inflammatory response, and decreased the proportion and number of Th2 and Th17 cells in asthmatic mice. Besides the increase in Th2 and Th17 cells, the findings indicate that G-MDSC elevation plays a crucial role in asthmatic mice.

## Introduction

Asthma is chronic airway inflammation characterized by airway hyperreactivity, mucus hypersecretion, and pulmonary inflammation. It is a complex immunological disorder caused by various immune cells and inflammatory factors. Th2 cells and type 2 cytokines, such as IL-4, IL-5, and IL-13, were initially identified as proinflammatory cells and factors in asthma 1-3. Subsequently, the imbalance of Th17 and Treg cells was reported to participate in the pathogenesis of asthma 4,5. Although Th2, Th17, and Treg cells, which are CD4^+^ T cells, are important in asthma pathogenesis, other innate and adaptive immune cells are reported to be involved in the complex inflammatory cascade in asthma, ultimately resulting in the asthma phenotype 6-8.

Myeloid-derived suppressor cells (MDSCs) are a heterogenous population with immunosuppressive activity and were initially identified in malignancies. MDSCs are originated from myeloid progenitor cells and immature myeloid cells. They are subdivided into granulocytic/neutrophilic MDSCs (G-MDSCs) and monocytic MDSCs (M-MDSCs). Recently, MDSCs were reported to play complex roles in asthma. CD11b^+^Gr-1^int^F4/80^+^ MDSCs accumulated in the lungs of asthmatic mice and suppressed lung DC-primed Th2 response 9. MDSCs were recruited to lung tissues by chemokine CCL2, and tumor-derived MDSCs can inhibit Th2-mediated inflammation in asthmatic mice 10. However, another study found that MDSCs promoted mast cell release inflammatory mediators, such as IL-6, TNF, and IL-13, ultimately leading to airway hyper-responsiveness and inflammation enhancement 11. The specific role and relationship of MDSCs with other immune cells, especially CD4^+^ T cells, in asthma are rarely reported.

In this study, we found imbalance of Th1/Th2 and Th17/Treg cells in experimental asthma mouse model. Asthmatic mice also showed an increased percentage of G-MDSCs, and increased mRNA expression of Th2- and Th17-related cytokines (IL-4 and IL-17). Th17 cell populations were closely related to the percentages of G-MDSCs. The reduction of MDSCs alleviated the inflammation, and decreased the proportion and number of Th2 and Th17 cells in asthmatic mice. The elevation of G-MDSCs and the presence of Th2 and Th17 cells may promote and maintain inflammatory response to asthma.

## Materials and Methods

### Animals and experimental design

Six- to eight-week-old male BALB/c mice (weighing 18-20g) were purchased from Comparative Medicine Centre of Yangzhou University, China, and maintained at the Animal Experimental Centre of Jiangsu University, China. All animals were given standard diet and water ad libitum under specific pathogen-free conditions. All animal experiments were approved by the Institutional Animal Care and Use Committee of Jiangsu University (Permit Number: JSU 16-127).

Mice were randomly assigned into one of three groups: normal, phosphate-buffered saline (PBS), and asthma. The asthma group was induced using an injection of 100 µg ovalbumin (OVA) (Sigma-Aldrich, Steinheim, Germany) and 2 mg of 10% aluminum hydroxide as adjuvant on days 1, 8, and 15, followed by 2% OVA intranasal challenges from day 22 to day 28. Mice were sacrificed within 24 h after the last challenge. Mice in the normal group were untreated, whereas those in the PBS group were sensitized and challenged with PBS alone.

### Measurement of OVA-specific serum IgE

The blood samples were centrifuged at 4000 rpm for 15 min. Then, the supernatants were collected for measurement of serum IgE by enzyme-linked immunosorbent assay. Briefly, the plates were coated with 100 µg/mL of OVA at 4 °C overnight in carbonate-bicarbonate buffer (pH 9.6). Then, the plates were washed with PBST and blocked with 5% skim milk. After washing, the wells were incubated with goat anti-mouse IgE (1:250, Abcam, Cambridge, MA, USA) for 2 h at 37 °C and then anti-goat secondary antibody (1:5000, Multisciences, Hangzhou, China). Finally, the reaction was terminated with sulfuric acid, and the OD value was measured at 450 nm.

### Cell counting in bronchoalveolar lavage fluid (BALF)

Mice were anesthetized after the last airway challenge, and alveolar lavage was performed after exposure of trachea. The lungs were washed thrice with 0.8 mL of cold PBS, and more than 90% of fluids were recovered. BALF was centrifuged at 800 rpm for 10 min. Then, the cell pellets were re-suspended with PBS for total cell count, and slides for eosinophil and neutrophil counting were prepared using Wright staining.

### Hematoxylin and eosin (H&E) staining

The left lung lobes were obtained and fixed with 10% neutral formalin and embedded in paraffin wax. Then, the fixed tissues were cut into sections (4 µm) and stained with H&E for airway inflammation analysis.

### Flow cytometry analysis

Splenocytes from mice were isolated as previously described 12. Single-cell suspensions of lungs were prepared from mice using the gentleMASCTM Octo Dissociator (Miltenyi Biotec, Bergish Gladbach, Germany). For MDSC analysis, single-cell suspensions from spleens and lungs were stained with FITC-anti-CD11b (cat. no.101205, BioLegend), APC-anti-Ly6G (cat. no. 127613, BioLegend), and PE-anti-Ly6C (cat. no. 126007, BioLegend). Moreover, Alexa Fluor 700-anti-Arginase 1 (cat. no. 56-3697-82, eBioscience, San Diego, CA, USA) and PE-Cyanine7-anti-iNOS (cat. no. 25-5920-80, eBioscience) were used for expression of MDSCs. For Th1, Th2, and Th17 cell subset analysis, spleen and lung cell suspensions were activated with PMA/Ionomycin Mixture (Multisciences, Hangzhou, China) and BFA/Monensin Mixture (MultiSciences) in a 5% CO2 incubator at 37 °C. After 5 h, the cells were stained with PerCP anti-CD3 mAbs (cat. no. 45-00310-80, eBioscience) and FITC-anti-CD4 (cat. no. 11-0041-82, eBioscience). Then, the cells were fixed and permeabilized with Cytofix/Cytoperm (BD Biosciences, San Jose, CA, USA). After washing with Perm/Wash buffer (BD Biosciences), the cells were stained with APC-anti-IFN-γ (cat. no. 17-7311-82, eBioscience), PE-anti-IL-4 (cat. no. 12-7041-82, eBioscience), and PE-anti-IL-17A (cat. no. 506904, BioLegend). For Treg cell analysis, the Mouse Regulatory T Cell Staining Kit (eBioscience) was used following the manufacturer's instructions. All stained cell samples were analyzed with a BD FACSCanto flow cytometer (BD Biosciences). Data were analyzed with FlowJo v10.0.7 software (Tree Star, Ashland, OR, USA). The absolute numbers of cells were calculated as: % cells from flow cytometry × total number of cells/100 13.

### Real-time quantitative PCR (RT-qPCR)

Total RNA was isolated from spleen cells and lungs by using TRIzol Reagent (Ambion, Austin, TX, USA) in accordance with the manufacturer's protocol. Total RNA (1 µg) was reverse-transcribed using All-in-One™ First-Strand cDNA Synthesis Kit (GeneCopoeia, Germantown, MD, USA) in accordance with the manufacturer's protocol. All-in-One™ qPCR primer sets for GAPDH (MQP027158), IFN-γ (MQP027401), IL-4 (MQP032451), IL-17A (MQP029457), TGF-β (MQP030343), and IL-10 (MQP029453) were purchased from GeneCopoeia. The following primers (Sangon Biotech, Shanghai, China) were used: IL-6 (forward: 5′- AGGAGTGGCTAAGGACCAAGACC -3′, reverse: 5′- CTGACCACAGTGAGGAATGTCCAC -3′). The samples were analyzed using RT-qPCR with BlazeTaq™ SYBR® Green qPCR Mix (GeneCopoeia) in accordance with the manufacturer's instructions. The samples were amplified at 95 °C for 30 s, followed by 40 cycles at 95 °C for 10 s and at 60 °C for 20 s, and then at 72 °C for 15 s.

### MDSC depletion

For the in vivo depletion of MDSCs, the mice were intraperitoneally injected with 60 mg/kg gemcitabine (Sigma-Aldrich) every 7 days from day 3 after the first OVA immunization. Gemcitabine has been confirmed as an effective substance that depletes of MDSC 14,15.

### Statistical analysis

Statistical analysis was performed with SPSS22.0 and GraphPad Prism 5.01 (GraphPad Software, 2007, La Jolla, CA, USA), and the results were presented as mean ± standard error of the mean. One-way analysis of variance together with the Tukey test was used to calculate the differences among three groups. The correlation was analyzed using Spearman correlation. Statistical significance was considered at *P* < 0.05.

## Results

### Inflammatory response to OVA-induced asthma in mice

**Figure 1:**
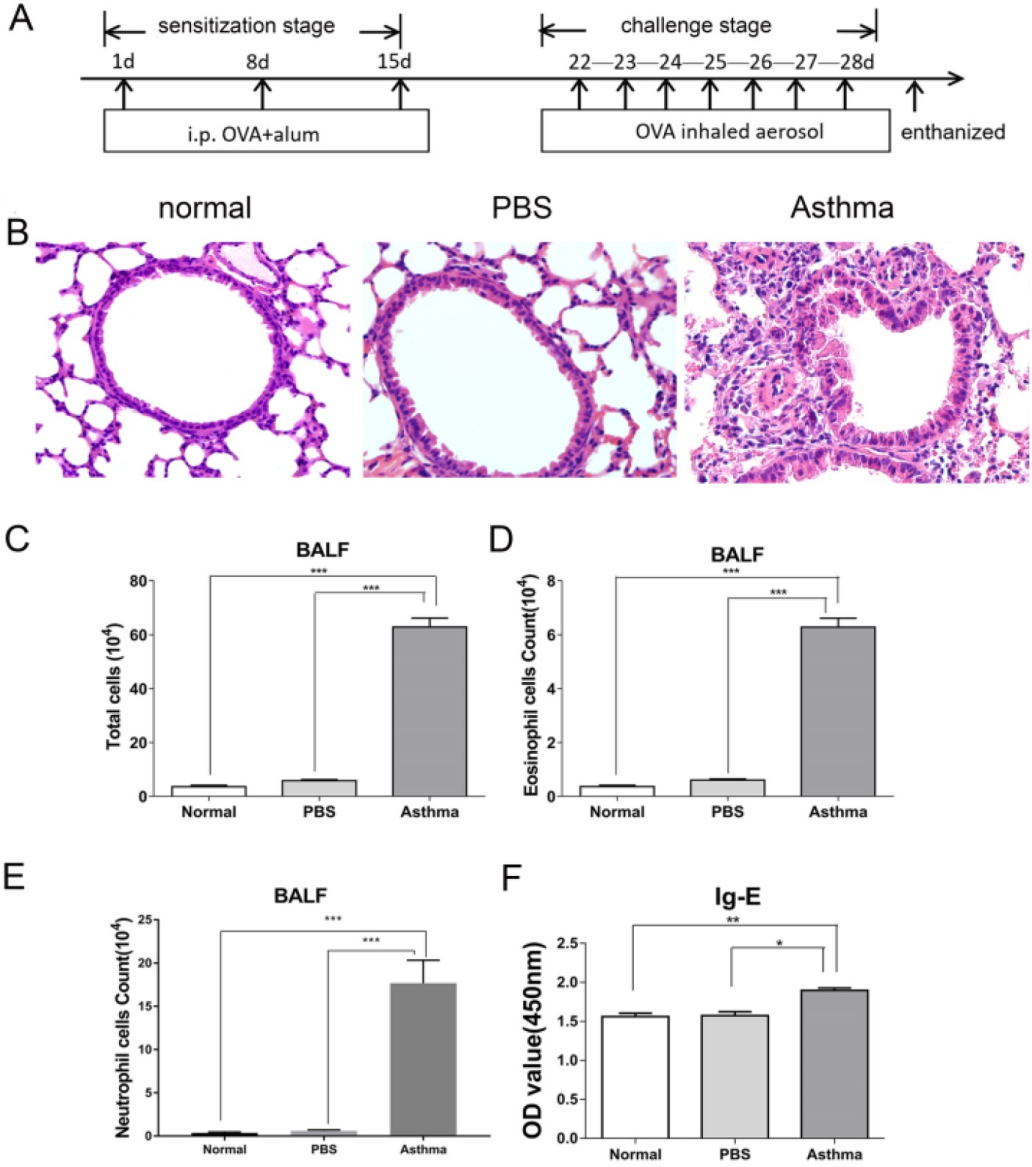
** OVA-induced inflammatory response in mice. (A)** Experimental scheme. Asthma group was induced by injection with 100 µg of OVA and 2 mg of 10% aluminum hydroxide as adjuvant on days 1, 8, and 15 and challenged by 2% OVA daily from day 22 to day 28. **(B)** Histological analysis of lung tissue by H&E staining (magnification 400×). **(C)** Total cells in BALF. **(D)** Number of eosinophils was calculated in BALF. **(E)** Number of neutrophils was calculated in BALF. **(F)** OVA-specific IgE level in sera of mice. The values are mean±SEM (n=12) from two independent experiments. *P < 0.05, **P < 0.01, ***P < 0.001.

Mice were immunized and challenged by OVA to establish experimental asthma. Figure 1A shows the treatment regimen. As shown in Figure 1B, asthmatic mice exhibited intense inflammatory cell infiltration and increased thickness and destruction of the alveolar wall and mucus secretion. Furthermore, mice in the asthma group had higher numbers of eosinophil, and neutrophil cells in BALF than those in the normal or PBS group (Figure 1C-E). Moreover, mice in the asthma group had higher levels of OVA-specific IgE compared with those in the normal or PBS group (Figure 1F). These results suggest that OVA-induced asthmatic mice displayed intense airway inflammatory response.

### Increased Th2 and Th17 cells and decreased Th1 and Treg cells in the splenocytes and lungs of asthmatic mice

**Figure 2:**
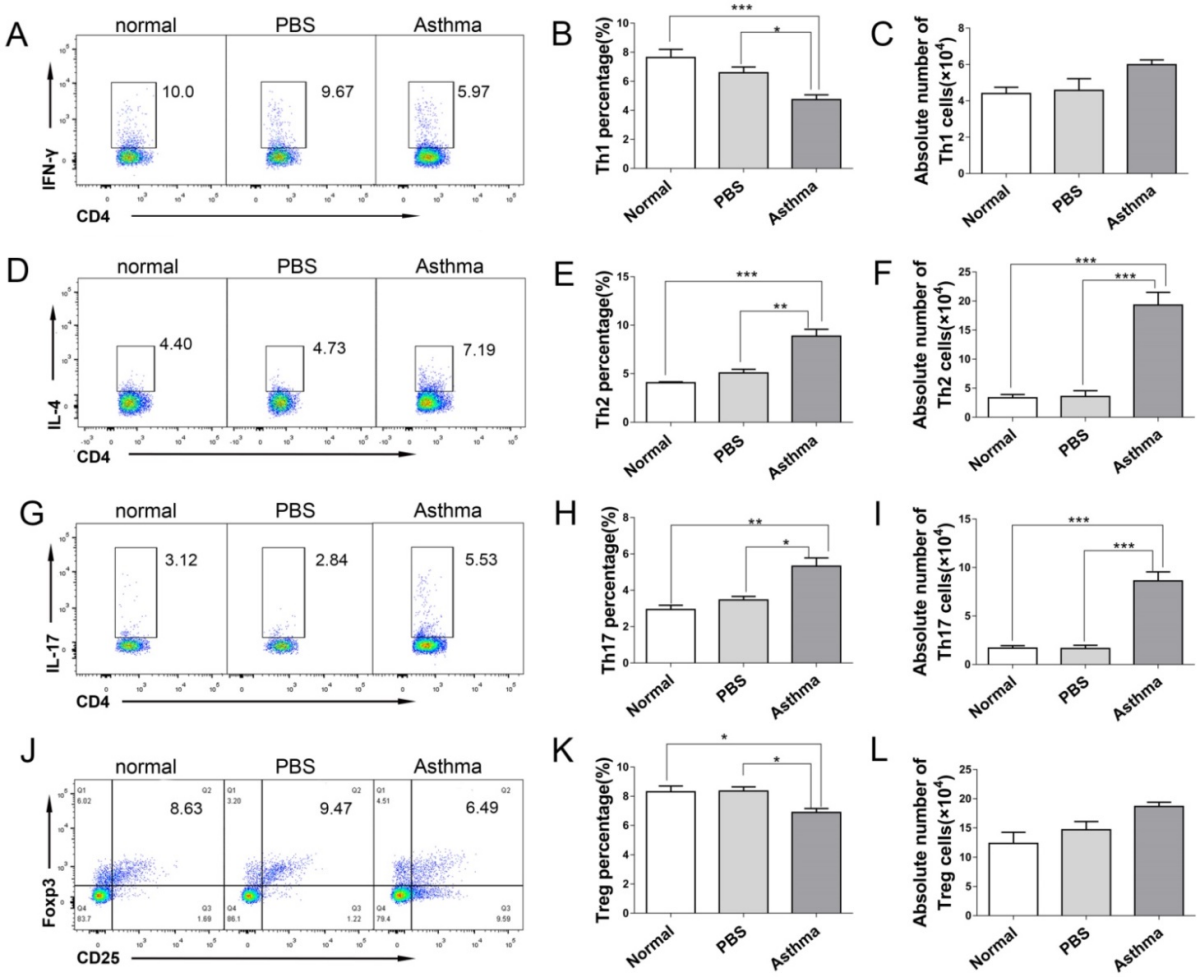
** Th1/Th2/Th17/Treg cell subset distribution in lungs of mice.** The single-cell suspensions of lungs in mice were detected for Th1/Th2/Th17/Treg cell subsets by flow cytometry. **(A)** CD4^+^IFN-γ^+^ Th1 cells,** (B)** Th1 cell percentage, **(C)** Absolute number of Th1 cells, **(D)** CD4^+^IL-4^+^ Th2 cells, **(E)** Th2 cell percentage, **(F)** Absolute number of Th2 cells, **(G)** CD4^+^IL-17^+^ Th17 cells, **(H)** Th17 cell percentage, **(I)** Absolute number of Th17 cells, **(J)** CD4^+^CD25^+^Foxp3^+^ Treg cells, **(K)** Treg cell percentage, and **(L)** absolute number of Treg cells each group are shown. The values are mean±SEM of 12 mice from two independent experiments. *P < 0.05, **P < 0.01, ***P < 0.001.

CD4^+^ T cells are the forefront of airway inflammatory response in asthma 7. To determine the role of CD4^+^ T cells during inflammatory response in asthmatic mice, we measured the frequency and absolute number of Th1, Th2, Th17, and Treg cells from the splenocytes and lungs of mice by using flow cytometry. Consistent with the results described previously in patients with allergy 15, the proportion and absolute number of CD4^+^IL-4^+^Th2 and CD4^+^IL-17^+^Th17 cells were significantly higher in splenocytes and lungs of asthmatic mice than in those from normal or PBS group. Conversely, the proportion and absolute number of CD4^+^IFN-γ^+^Th1 in splenocytes were significantly lower in asthmatic mice than in the mice in the normal or PBS group (Figures S1D-I, and Figures 2D-2I). However, the proportion of CD4^+^CD25^+^Foxp3^+^ Treg cells in the spleen and lungs of asthmatic mice decreased, but their absolute number did not decrease (Figures S1J-1L, and Figures 2J-2L). These results suggested an imbalance of Th1/Th2 and Th17/Treg cells in asthmatic mice.

### Increased the number of MDSC in the splenocytes and lungs of asthmatic mice

**Figure 3:**
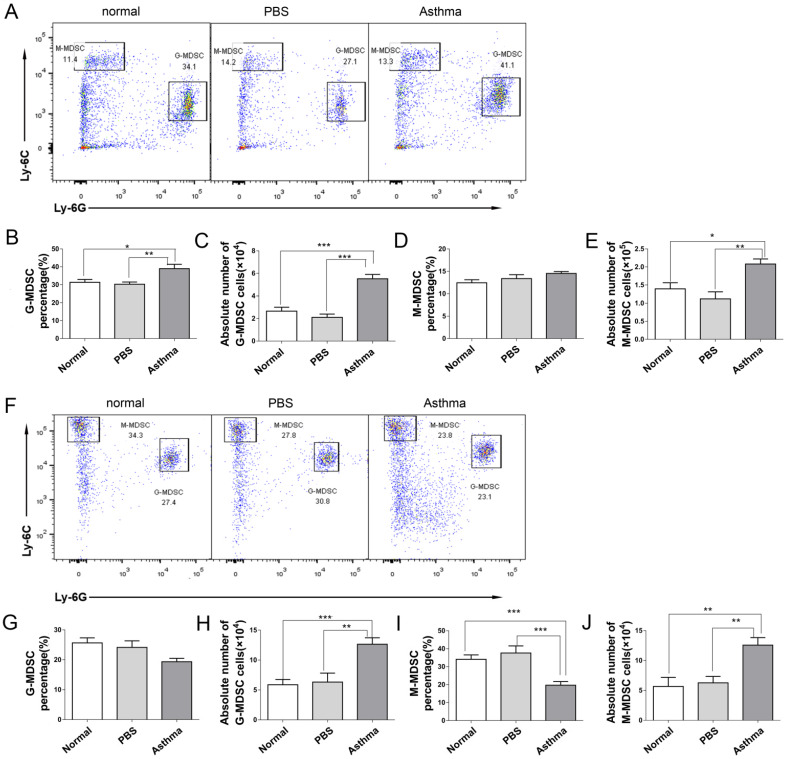
** MDSC subset distribution in splenocytes and lungs of mice.** The splenocytes and the single-cell suspensions of lungs in mice were tested for CD11b^+^Ly6G^-^Ly6C^high^ M-MDSC and CD11b^+^Ly6G^+^Ly6C^low^ G-MDSC subsets by flow cytometry. (A) Representative flow cytometry results are shown in splenocytes of mice. (B) G-MDSC percentage, (C) Absolute number of G-MDSCs, (D) M-MDSC percentage and (E) Absolute number of M-MDSCs in splenocytes of each group are shown. (F) Representative flow cytometry results are shown in lungs of mice. (G) G-MDSC percentage, (H) Absolute number of G-MDSCs, (I) M-MDSC percentage and (J) Absolute number of M-MDSCs in lungs of each group are shown. The values are mean±SEM of 12 mice from two independent experiments. *P < 0.05, **P < 0.01, ***P < 0.001.

Recent studies have demonstrated that MDSCs have a complex role in asthma 17,18. To investigate the generation of MDSCs during an inflammatory response in asthmatic mice, we measured the frequency and absolute number of two subsets of MDSCs from the splenocytes and lungs of mice via flow cytometry. M-MDSCs defined as CD11b^+^Ly6G^-^Ly6C^high^ and G-MDSCs defined as CD11b^+^Ly6G^+^Ly6C^low^ 19. As shown in Figure 3, the proportion and absolute number of G-MDSCs in the splenocytes of asthmatic mice were higher than those in the mice of the normal or PBS group (Figures 3B and 3C). However, the absolute number of M-MDSCs in the splenocytes of asthmatic mice increased compared with those in the normal or PBS group, but the proportion of M-MDSCs did not increase (Figures 3D and 3E). The percentage of M-MDSCs in the lungs of asthmatic mice decreased, but the absolute number of G-MDSCs and M-MDSCs increased (Figures 3G-3J). Thus, the MDSC level of asthmatic mice increased.

### Elevated expression of pro-inflammatory cytokines in splenocytes and lungs of asthmatic mice

**Figure 4:**
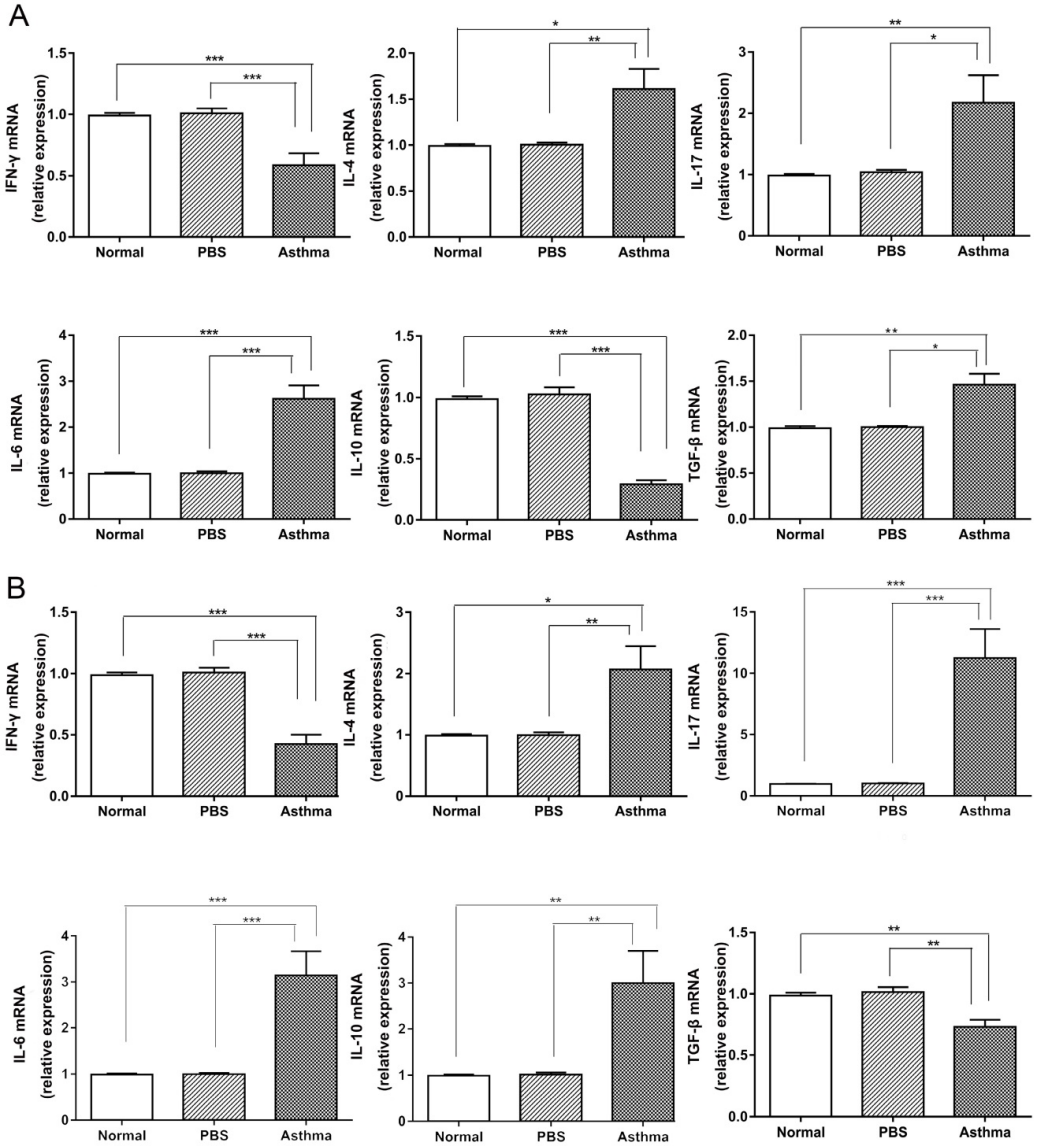
** Cytokine expression in the spleen and lung tissue of mice.** (A) The mRNA expression levels of IFN-γ, IL-4, IL-17A, IL-6, IL-10, and TGF-β in the spleen were evaluated by RT-qPCR. (B) The mRNA expression levels of IFN-γ, IL-4, IL-17A, IL-6, IL-10, and TGF-β in lung tissue were measured through RT-qPCR. The values are mean±SEM of 12 mice from two independent experiments. *P < 0.05, **P < 0.01, ***P < 0.001.

Cytokines produced by immune cells and bronchial epithelial cells shape the phenotypic and pathological features of asthma 6. Thus, we detected the expression of cytokines in the splenocytes and lungs of mice. Consistent with the results of Th cell subset in mice (Figure S1 and Figure 2), the asthma group displayed higher levels of IL-4, IL-17, IL-6, and TGF-β mRNA expression and lower levels of IFN-γ and IL-10 mRNA in the splenocytes than the normal and PBS groups (Figure 4A). However, higher levels of IL-4, IL-17, IL-6, and IL-10 mRNA and lower levels of IFN-γ and TGF-β mRNA were observed in lungs of mice from the asthma group relative to those from the normal or PBS group (Figure 4B). These results suggest that asthmatic mice produced proinflammatory and T-cell-dependent cytokines in the immune system and inflammatory site.

### Relationship of G-MDSCs, Th17 cells, and Treg cells in asthmatic mice

**Figure 5:**
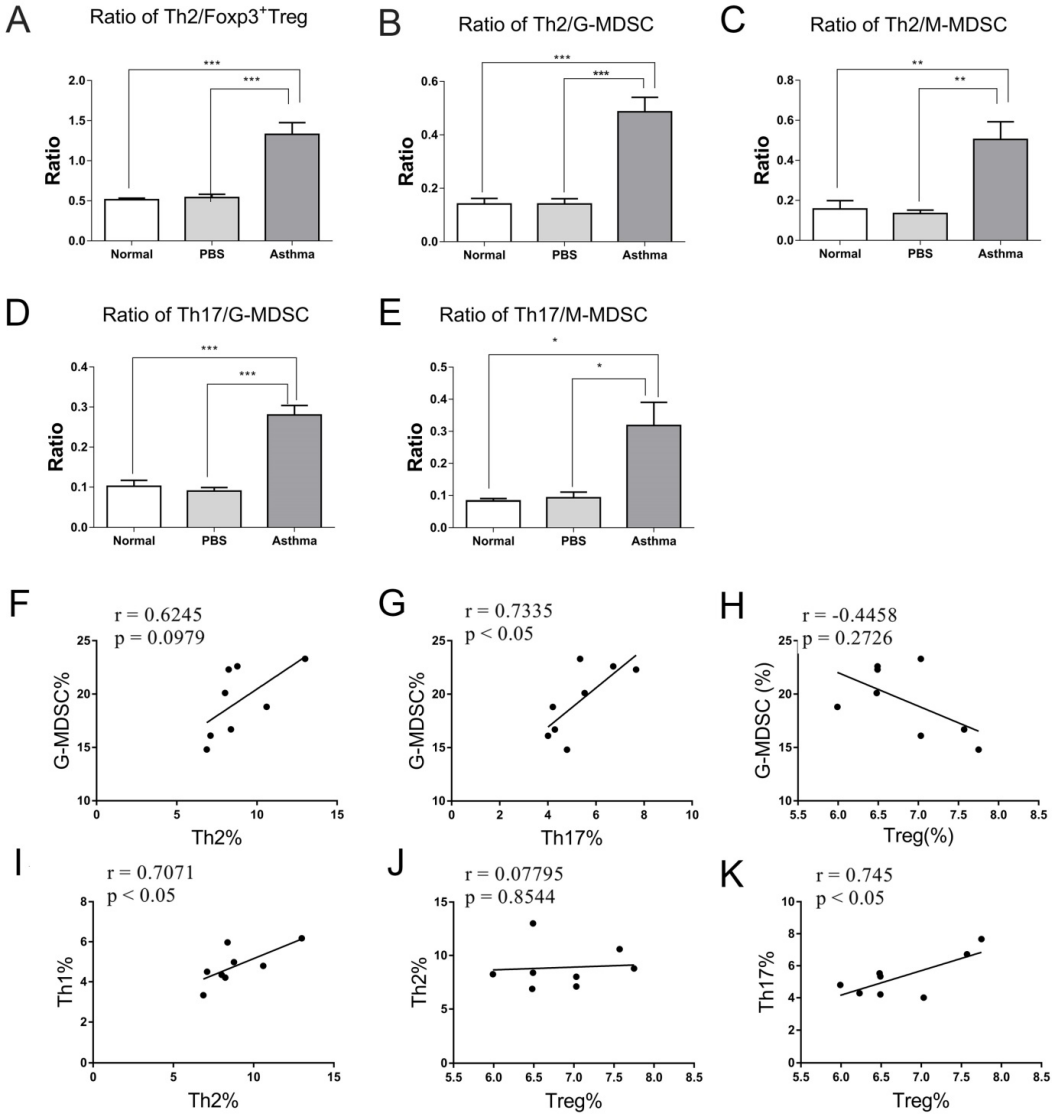
** Ratios and relationships among Th1, Th2, Th17, Treg, G-MDSC, and M-MDSC in mice. (A)** Ratios of Th2/Treg, **(B)** Th2/G-MDSC, **(C)** Th2/M-MDSC, **(D)** Th17/G-MDSC, and **(E)** Th17/M-MDSC in each group are shown. The values are mean±SEM of 12 mice from two independent experiments. *P < 0.05, **P < 0.01, ***P < 0.001. **(F)** Relationship of G-MDSCs with Th2 cells, **(G)** G-MDSCs with Th17 cells, and **(H)** G-MDSCs with Treg cells in asthmatic mice. **(I)** Relationship of Th1 cells with Th2 cells, **(J)** Th2 cells with Treg cells, and **(K)** Th17 cells with Treg cells in asthmatic mice (n=8).

Given the inflammatory sites is lung in asthmatic mice, which was mediated by high levels of Th2 cells, Th17 cells, and MDSCs and low levels of Treg cells in asthmatic mice. Thus, we further compared the ratio of effector T cells to Treg cells and MDSCs in lungs of mice and analyzed their correlation. Consistent with the analysis of Th cell subset in Figure S1 and Figure 2, the ratio of Th1/Th2 cells was significantly decreased in the asthma group than in the normal or PBS group (data not shown). However, the asthma group displayed higher Th2/Treg, Th2/G-MDSC, Th2/M-MDSC, Th17/G-MDSC, and Th17/M-MDSC ratio in lungs relative to the normal or PBS group (Figures 5A-C). Furthermore, the percentage of Th17 was positively correlated with that of G-MDSC and Treg cells in lungs of asthmatic mice (Figures 5G and 5K). However, no correlation was observed between Th2 cells and Treg cells or G-MDSCs (Figures 5F and 5H). By contrast, the percentage of Th1 cells was positively correlated with that of Th2, and the percentage of Th17 cells was positively correlated with that of Treg cells in lungs of asthmatic mice (Figures 5I and 5K). These results suggest a close correlation among G-MDSCs, Th17 cells, and Treg cells, which may together maintain the inflammatory response in asthma.

### Alleviation of inflammation in asthmatic mice via MDSCs depletion

**Figure 6:**
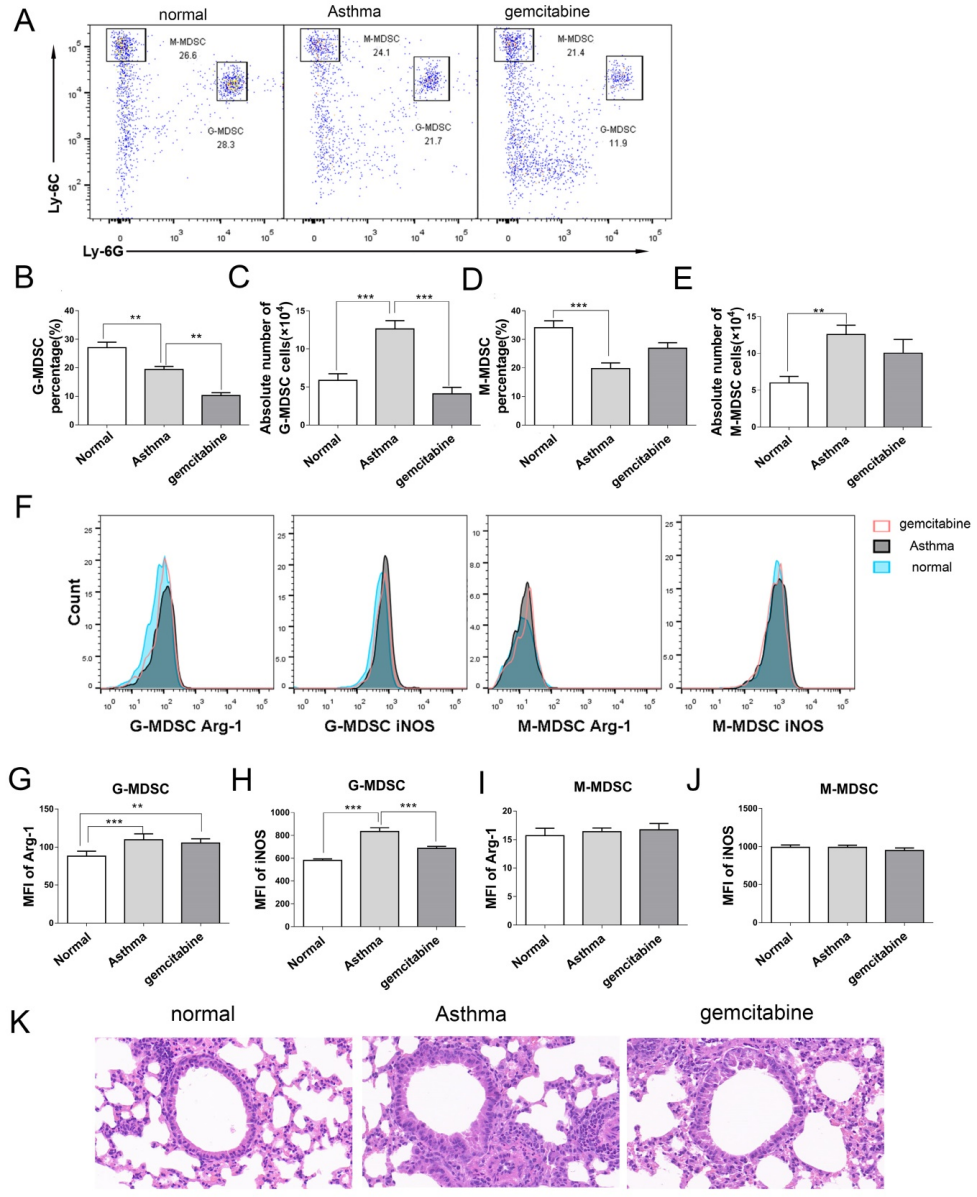
** Reduction number of MDSCs alleviates inflammation in asthmatic mice.** Asthma was induced in mice that were then treated with gemcitabine as described in the Materials and Methods section. The single-cell suspensions of the lungs in mice were tested in terms of CD11b^+^Ly6G^-^Ly6C^high^ M-MDSC and CD11b^+^Ly6G^+^Ly6C^low^ G-MDSC subsets via flow cytometry. Arg-1 and iNOS expression levels were gated from G-MDSCs and M-MDSCs, respectively. **(A)** Representative flow cytometry results are shown. **(B)** The percentage and** (C)** absolute number of G-MDSCs and the percentage and **(E)** absolute number of M-MDSCs in each group are shown. **(F)** Arg-1 and iNOS expression levels in G-MDSCs and M-MDSCs are shown. **(G)** MFI of Arg-1 in G-MDSCs**, (H)** MFI of iNOS in G-MDSCs, **(I)** MFI of iNOS in M-MDSCs, and **(J)** MFI of iNOS in M-MDSCs in each group are shown. *P < 0.05, **P < 0.01, ***P < 0.001. **(K)** Histological analysis of the lung section from mice via H&E staining (20× magnification). Images are representatives of two independent experiments (n = 6 mice per group).

To determine the role of MDSCs in asthma, we used gemcitabine to deplete endogenous MDSCs without affecting other immune cells, including T cells, B cells, and dendritic cells 20,21. In Figures 6A-6E, gemcitabine treatment significantly decreased the percentage and absolute number of G-MDSCs in the lungs of mice, but did not affect M-MDSCs compared with those of the asthma group. Furthermore, G-MDSCs expressed Arg-1 and iNOS, whereas M-MDSCs only expressed iNOS (Figure 6F). The mean fluorescence intensity (MFI) of Arg-1 and iNOS from G-MDSCs in the asthma group was higher than that in the normal group (Figures 6G and 6H). Gemcitabine treatment significantly decreased the MFI of iNOS from G-MDSCs compared with that in the asthma group (Figures 6H and 6J). Thus, gemcitabine treatment reduced the number of G-MDSCs and the level of iNOS expression in asthmatic mice. Furthermore, the infiltration of inflammatory cell and the thickness and destruction of the alveolar wall in gemcitabine-treated mice were alleviated compared with those in the asthma group (Figure 6K). These results suggested that G-MDSCs accumulated in asthmatic mice and induced an inflammatory response. The reduction of G-MDSCs alleviated the pathological processes in asthmatic mice.

### Decreased Th2 and Th17 cells in the lungs of asthmatic mice via MDSC depletion

**Figure 7:**
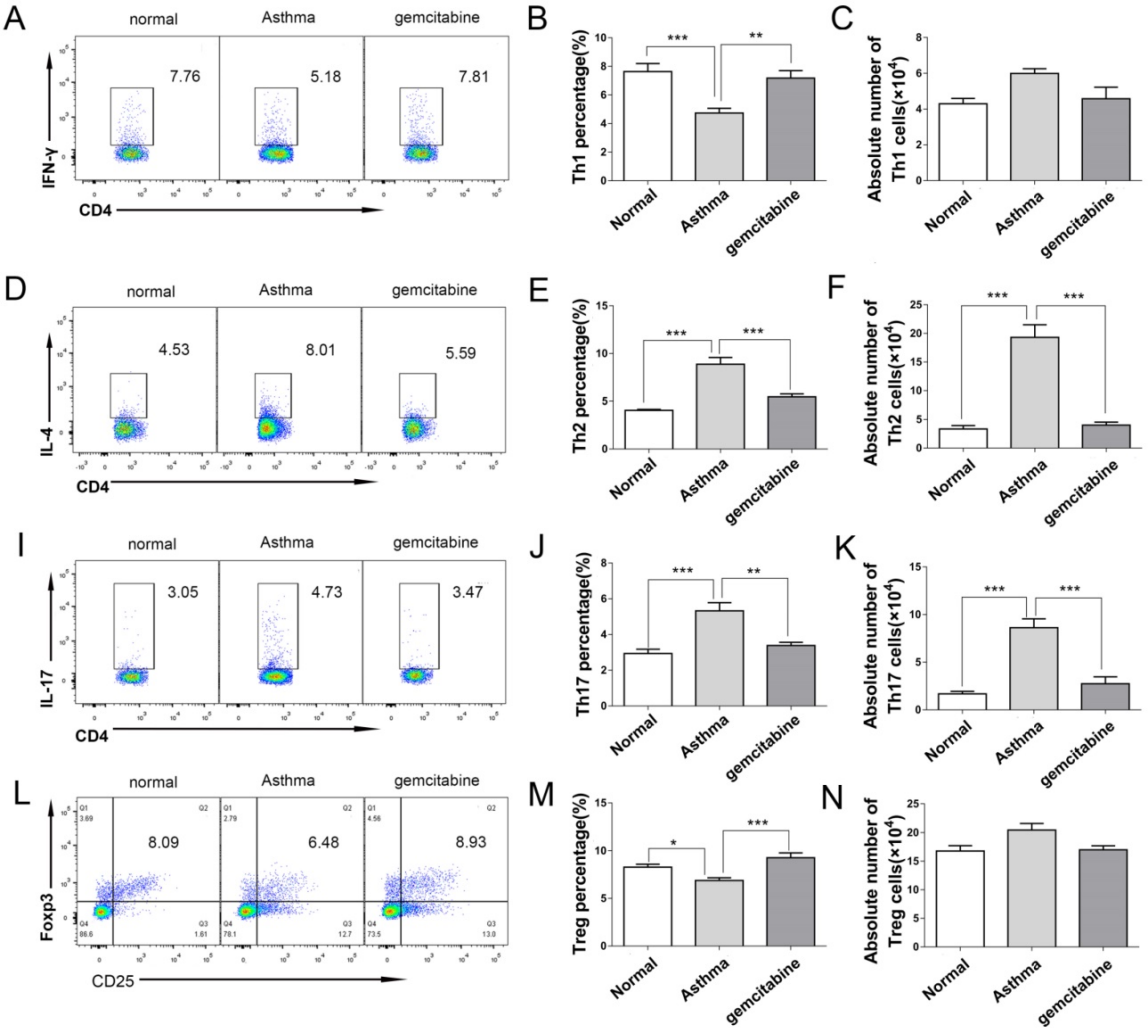
** Depletion of MDSCs reduces the number of Th2 and Th17 cells in asthmatic mice.** Asthma was induced in mice that were then treated with gemcitabine as described in the Materials and Methods section. The single-cell suspensions of the lungs in mice were tested for Th1/Th2/Th17/Treg cell subsets by flow cytometry. **(A)** CD4^+^IFN-γ^+^ Th1 cells, **(B)** Th1 cell percentage, **(C)** Absolute number of Th1 cells, **(D)** CD4^+^IL-4^+^ Th2 cells, **(E)** Th2 cell percentage, **(F)** Absolute number of Th2 cells, **(I)** CD4^+^IL-17^+^ Th17 cells, **(J)** Th17 cell percentage, **(K)** Absolute number of Th17 cells, **(L)** CD4^+^CD25^+^Foxp3^+^ Treg cells, **(M)** Treg cell percentage, and **(N)** absolute number of Treg cells each group are shown. The values are mean±SEM of 12 mice from two independent experiments. *P < 0.05, **P < 0.01, ***P < 0.001.

To further investigate the role of MDSCs on Th distribution in asthma, we detected the Th cells in the lungs of asthmatic mice after MDSC depletion by gemcitabine. Consistent with the results of Th in Figure 2, Figure 7 reveals that the proportion and absolute numbers of CD4^+^IL-4^+^Th2 and CD4^+^IL-17^+^Th17 cells were significantly higher in the asthmatic mice than in the normal mice. By contrast, the percentages of CD4^+^IFN-γ^+^Th1 and CD4^+^CD25^+^Foxp3^+^ Treg were significantly lower in the asthmatic mice than in the normal mice (Figure 7). However, gemcitabine treatment decreased the proportion and number of Th2 and Th17 cells but increased the proportion of Th1 and Treg cells in asthmatic mice (Figure 7). Thus, the reduction of Th2 and Th17 cells induced by MDSC depletion might alleviate inflammatory responses in allergic mice.

## Discussion

Asthma is a complex and heterogeneous inflammatory disease. Many environmental factors and immune cells are responsible for asthma, making its pathogenesis complex. Apart from the Th2 response in asthma, Th1, Th17, Treg, and other innate immune cells, including MDSCs, are involved in the pathological response of asthma. Thus, understanding the heterogeneity of pathogenesis of asthma may offer new therapeutic strategies for this severe public problem 6.

In the present study, asthmatic mice showed increased Th2 and Th17 cells and decreased Th1 and Treg cells, which is consistent with a previous study 22. Apart from the Th cell subset, MDSCs were also increased in asthmatic mice. MDSCs have been reported to participate in the inflammatory response in autoimmunity and cancer through regulating innate and adaptive immune responses 23. Asthma is a chronic airway inflammation elicited by various cells and mediators. Thus, increased MDSCs may also contribute to the pathogenesis of asthma. These results are consistent with those of a previous study that reported the accumulation of MDSCs in asthmatic children and mice, which may be involved in the development of asthma 24. However, Shi et al. reported that CD11b^+^Gr-1^high^Ly6G^+^Ly6C^int^ polymorphonuclear MDSCs (PMN-MDSCs) accumulated in the lungs of asthmatic mice and were negatively correlated with airway inflammation 25. Guan et al. found that CD45^+^CD33^+^CD14^+^HLA-DR^-/low^CD15^-^ M-MDSC decreased in WBCs of asthma patients than those of healthy controls, whereas CD45^+^CD33^+^CD14^-^CD15^+^CD66b^+^ PMN-MDSCs showed no difference between asthma patients and controls 26. Kolahian and coworkers found that neutrophilic MDSCs (PMN-MDSCs) number decrease in BALF and lungs of asthmatic mice, and adoptively transferred PMN-MDSCs can inhibit Th2-domminant inflammation in the lungs of mice. Asthma in these mice was induced by OVA sensitization and house dust mouse (HDM) challenge 27. Future studies should investigate whether these differences are due to the different disease microenvironments caused by various modeling method. Deshane et al. identified three subsets of CD11b^+^Ly6G^+^ myeloid cells that infiltrated the lungs of asthmatic mice. They demonstrated that the Ly6G^-^Ly6C^+^ and Ly6G^+^Ly6C^+^ subsets could suppress T-cell proliferation, whereas the Ly6G^+^Ly6C^-^ subset promoted T-cell responses and exacerbated airway inflammatory response 28. Although we did not investigate the effect of MDSCs on T cells, we found that the absolute numbers of CD11b^+^Ly6G^+^Ly6C^low^ G-MDSCs and CD11b^+^Ly6G^-^Ly6C^high^ M-MDSCs were increased in splenocytes and lungs of asthmatic mice. Thus, increased MDSCs may play an important role in asthmatic mice by affecting T cells, especially Th2, Th17, or Treg cell response in asthma.

In line with the results of Th subsets in asthmatic mice (Figure S1and 2), the expression levels of IL-4, IL-17, and IL-6 gene were high, and those of IFN-γ gene were low in splenocytes and lungs of asthmatic mice. However, asthmatic mice displayed divergence in gene expression of IL-10 and TGF-β from splenocytes and lungs. IL-10 and TGF-β are produced by various immune cells in splenocytes and released by immune and stromal cells in the lungs 29. In this study, the expression of IL-10 was decreased in the splenocytes of asthmatic mice, which is consistent with reduced airway IL-10 levels and IL-10 release by CD4^+^ T cells in pediatric asthmatics 30. Consistent with these findings, Kianmehr et al. also found that TGF-β expression was increased in the splenocytes of asthmatic mice 22. The production of cytokines in the lungs is more complex than that of splenocytes because immune cells cross-talk with stromal cells, which together induce complex cytokine levels 29. Thus, T-cell subsets not only often present a mixed phenotype, but other proinflammatory cells are present in asthma because of the plentiful cytokines in the inflamed lung 7. These cytokines mediate cell-cell communication, which would control pulmonary immunity, mediating the chronic inflammatory response of the airways in asthma 29.

MDSCs are reported to regulate T-cell response and mediate Treg expansion in autoimmunity 31. In an asthmatic mouse model, MDSCs recruited Treg cells and suppressed T-cell response 28. However, in adult atopic asthmatic models, robust expansion of MDSCs was observed in BALF after allergen challenge, and these MDSCs were associated with Th2 response and IL-10 production 32. Consistent with the results in asthma patients 26, the ratio of Th2/Treg was increased in asthmatic mice relative to mice in the normal and PBS group. Inconsistent with a study that found increased Th2/M-MDSC and Th17/M-MDSC in asthma patients 26, we observed increased ratios of Th2/G-MDSC, Th2/M-MDSC, Th17/G-MDSC, and Th17/M-MDSC in asthmatic mice. Furthermore, in contrast to the positive correlation of Th2 with M-MDSC in asthma patients 26, we did not find a correlation between Th2 and G-MDSCs or M-MDSCs in asthmatic mice. However, we demonstrated that Th17 cells are positively correlated with G-MDSCs (Figure 7), and no correlation was observed between M-MDSCs and Th2 or Th17 cells in asthmatic mice (data not shown). This difference between humans and mice might be related to differences in identification of MDSCs, genetic background, and pathogenesis between mice and humans. This finding also indicates that the mouse model does not completely mimic the complexity of human asthma.

Numerous studies have reported the association between MDSCs and Th17 cells in autoimmune disease. Jiao et al. reported that the frequency of circulating MDSCs and Th17 cells and ARG-1 levels increased in patients with rheumatoid arthritis, and Th17 cells were negatively correlated with MDSC percentage and plasma ARG-1 levels 33. G-MDSCs can inhibit Th17 response and mitigate disease severity in experimental autoimmune encephalomyelitis (EAE) 34, but CD11b^+^Gr-1^+^ MDSCs also have been reported to facilitate Th17 cell differentiation and promote the development of EAE in mice 35. In addition, circulating MDSCs and Th17 cells were elevated in esophageal cancer patients, and MDSCs were correlated with IL-17 levels in gastrointestinal cancer patients 36. Numerous studies also reported that MDSCs mediate Treg cell expansion 37,38. However, G-MDSCs derived from tumors hinder the amplification of natural Treg cells, weaken the TGF-β1-mediated generation of induced Treg (iTreg) cells, and suppress naïve CD4^+^ T-cell differentiation into iTreg cells 39. Thus, different subsets of MDSCs may orchestrate the differentiation of the effector or regulatory T-cell subsets derived from naïve CD4^+^ T cells and these subsets can shape the immune response from inflammation to tolerance. Asthma is a chronic inflammation. Whether the increase of G-MDSCs promotes Th17 differentiation while inhibiting Treg cell differentiation, resulted in increased Th17 cells and decreased Treg cells in asthmatic mice, needs further analysis.

Consistent with previous reports that gemcitabine treatment reduces the number of MDSCs in mice 14-15;20-21; our study demonstrated that gemcitabine injection mainly decreased the G-MDSC level in asthmatic mice. Furthermore, gemcitabine treatment not only decreased the G-MDSC level but also inhibited the iNOS expression from G-MDSCs in asthmatic mice. MDSCs reduction by gemcitabine expectedly impaired inflammation and decreased the proportion and number of Th2 and Th17 cells in asthmatic mice. In general, iNOS generates nitric oxide (NO) from L-arginine 40, resulting in an increased pro-inflammatory NO 41. Myeloid regulatory cells, including MDSCs, produce NO, IL-6, IL-1β and IL-23, which induce Th17 cells differentiation from naïve CD4 cells 35.Gemcitabine-induced decrease in G-MDSC number and iNOS expression may inhibit inflammatory responses in asthmatic mice by suppressing Th17 cell development, but this speculation should be further investigated. Gemcitabine was also reported to directly suppress CD4 T cell activation in EAE 42. Thus, the effect of gemcitabine on Th cells in asthmatic mice also needs further investigation. Moreover, gemcitabine primarily reduces G-MDSC levels in asthmatic mice, and it most likely induces apoptosis and cell death of the MDSC subset; the distinct patterns are dependent on effector type, a scenario similar to the effect of gemcitabine on Th cells in EAE 42. Gemcitabine promoted more Th17 and Treg cell apoptosis than Th1 cells in EAE, as Th17 cells can enter the central nervous system (CNS) more efficiently and generate more lesions than Th1 cells 43. Gemcitabine mainly decreased the G-MDSC levels. We can further deduce that G-MDSCs, but not M-MDSCs, promote inflammatory responses in asthmatic mice.

In summary, we demonstrated that asthmatic mice had increased levels of Th2, Th17, and MDSCs and decreased levels of Th1 and Treg cells in splenocytes and lungs. G-MDSCs were closely related to Th17 cells in asthmatic mice. MDSC Depletion alleviated inflammation and decreased the proportion and number of Th2 and Th17 cells in asthmatic mice. Thus, the increase in G-MDSCs and the presence of Th2 and Th17 cells can promote and maintain inflammatory responses in asthmatic mice.

## Supplementary Material

Supplementary figures and tables.
