# Supplementary material for: Elevated granulocytic myeloid-derived suppressor cells are closely related with elevation of Th17 cells in mice with experimental asthma
Source: Int J Biol Sci. 2020 May 16;16(12):2072–83. doi: 10.7150/ijbs.43596 (PMC7294949; doi:10.7150/ijbs.43596)
Supplement: Supplementary file 1 — Supplementary figures and tables. [file ijbsv16p2072s1.pdf]

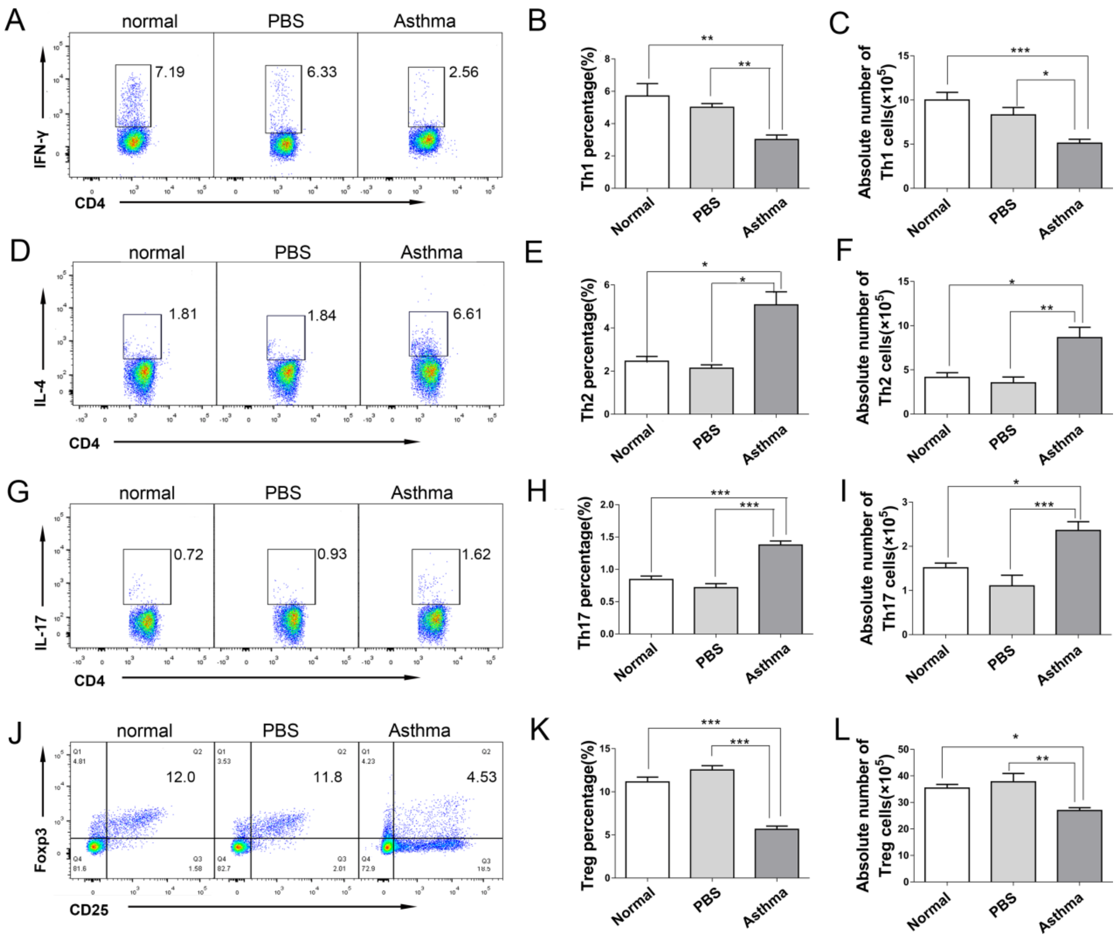

Figure S1. Th1/Th2/Th17/Treg cell subset distribution in splenocytes of mice. The splenocytes of mice were detected for Th1/Th2/Th17/Treg cell subsets by flow cytometry. (A) CD4$^+$IFN-γ$^+$ Th1 cells, (B) Th1 cell percentage, (C) Absolute number of Th1 cells, (D) CD4$^+$IL4$^+$ Th2 cells, (E) Th2 cell percentage, (F) Absolute number of Th2 cells, (G) CD4$^+$IL17$^+$ Th17 cells, (H) Th17 cell percentage, (I) Absolute number of Th17 cells, (J) CD4$^+$CD25$^+$Foxp3$^+$ Treg cells, (K) Treg cell percentage, and (L) absolute number of Treg cells in each group are shown. The values are mean±SEM of 12 mice from two independent experiments. *$P < 0.05$, **$P < 0.01$, ***$P < 0.001$.
